# Analysis of the Correlation between Frailty Index, Clinical Characteristics, Use of Anti-Epileptic Drug, and Prognosis in Elderly Patients with Epilepsy

**DOI:** 10.62641/aep.v53i2.1729

**Published:** 2025-03-05

**Authors:** Jianzhong Wang, Hengzhang Ma, Xiaodan Lin, Lixian Li, Zhixiong Zheng, Xiaohua Huang

**Affiliations:** ^1^Department of Neurology, Affiliated Mindong Hospital, Fujian Medical University, 355000 Fu’an, Fujian, China; ^2^Department of Geriatrics, Affiliated Mindong Hospital, Fujian Medical University, 355000 Fu’an, Fujian, China

**Keywords:** frailty index, epilepsy in the elderly, anti-epileptic drugs, degree of epileptic seizures

## Abstract

**Background:**

Epilepsy is a common neurological disorder among the elderly, often leading to significant morbidity. Therefore, it is necessary to study the correlation between the frailty index, clinical characteristics of epilepsy, use of anti-epileptic drug, and the prognosis of elderly patients with epilepsy.

**Methods:**

This retrospective study included 106 elderly patients with epilepsy who were treated at the Affiliated Mindong Hospital, Fujian Medical University, China, between January 2018 and December 2022. Based on the severity of the prognosis, the seizures were classified into the major seizure group (tonic-clonic), minor seizure group (absence, myoclonus, clonus, tonic, atonic and partial seizures), and no seizure group. Furthermore, the relationship between the frailty index, clinical characteristics, use of epilepsy drugs, and the degree of epileptic seizures was assessed using the Logistic regression analysis.

**Results:**

Univariate analysis indicated that older age (*p* < 0.001), longer disease duration (*p* = 0.009), and the presence of comorbid conditions such as diabetes (*p* = 0.002) and coronary heart disease (*p* < 0.001) were all associated with seizure severity. Additionally, frailty was significantly related to seizure severity, with the non-frailty group having fewer major seizures compared to the pre-frailty and frailty groups (*p* < 0.001). Similarly, regular medication use (*p* < 0.001) and the number of drugs taken (*p* < 0.001) were significant factors, with irregular medication use and single-drug regimens being more common in patients with more severe seizures. Multivariate Logistic regression analysis indicated that a higher frailty index (*p* = 0.033), age over 70 years (*p* = 0.015), longer disease duration (*p* = 0.003), the presence of coronary heart disease (*p* < 0.001), and regular medication use (*p* = 0.022) were all significantly associated with more severe seizures.

**Conclusion:**

Frailty index, age, disease duration, coronary heart disease, and regular medication are related to the prognosis of elderly patients with epilepsy. These findings highlight the significance of comprehensive management strategies to improve clinical outcomes in this group of patients.

## Introduction

Epilepsy is a neurological disorder characterized by recurrent severe convulsions [1, 2]. In elderly patients, the causes of epilepsy are often secondary, with common causes including cerebral thrombosis, cerebral hemorrhage, brain tumor, brain atrophy, intracranial infection, brain trauma, neurodegenerative diseases, and autoimmune conditions [3]. The diverse etiology of epilepsy in elderly patients leads to complex pathophysiology and clinical heterogeneity, making it challenging to evaluate the prognosis and treatment response. Furthermore, elderly patients often have multiple comorbidities and take various medications, increasing the risk of drug interactions. Additionally, their increased sensitivity to anti-epileptic drugs, along with a reduced glomerular filtration rate, can lead to prolonged drug retention in their body. Therefore, selecting medications that do not rely on renal filtration is crucial to ensure efficacy and safety for a favorable prognosis [4].

The “Frailty Index” is a crucial indicator of the biological health of the elderly [5]. It is calculated based on the principle of accumulating individual health defects by dividing the number of health criteria an individual meets by the total number of criteria included. This index serves as an indicator to assess the degree of aging in an individual [6, 7]. Currently, there are no reports correlating the frailty index to the prognosis of epilepsy in elderly patients. Furthermore, there are no detailed clinical guidelines for diagnosing and managing epilepsy in elderly patients, particularly concerning clinical characteristics and the use of anti-epileptic drugs.

Therefore, this study aims to explore the relationship between the frailty index, clinical characteristics, the use of anti-epileptic drugs, and the clinical prognosis in elderly epileptic patients. The findings intend to provide clinicians with a theoretical basis to accurately formulate patient treatment plan.

## Data and Methods

### Study Participants

This retrospective study included 106 elderly epileptic patients treated at the Affiliated Mindong Hospital, Fujian Medical University, China, between January 2018 and December 2022. This study received approval from the ethics committee of the Affiliated Mindong Hospital, Fujian Medical University, China (2024051005), and was conducted following the principles of the Declaration of Helsinki. Furthermore, written informed consent was obtained from all participants.

Inclusion criteria included patients who met the 2015 diagnostic criteria for epilepsy established by the Chinese Anti-Epilepsy Association [8], patients aged between 60 and 75 years, duration of anti-epileptic drug treatment ≥6 months, and those taking one or more anti-epileptic drugs.

However, the exclusion criteria were set as follows: patients aged <60 years or >75 years; those in perimenopause, postmenopause, pregnancy or lactation; patients with organic lesions in vital organs; those with an abnormal coagulation system; patients with mental disorders who are unable to care for themselves, and those with incomplete clinical case data.

### Frailty Index

Based on the cumulative defect model proposed by Rockwood and Mitnitski [9], when negative factors are greater than positive factors, the frailty index will increase. This includes the number and severity of the current disease, the ability to perform daily life activities, and the symptoms and signs found during physical or neurological assessments. The frailty index determines a patient’s frailty by computing the ratio of health-related problems to the total number of assessed items. The fateful assessment scale includes 34 variables [10], such as health status, daily living abilities, instrumental daily living abilities, chronic diseases, and clinical symptoms. For each occurrence of a health-related item, 1 point is recorded, and 0 points are recorded if there are no relevant events. However, factors like visual impairment, hearing impairment, physical pain, and self-rated health status are scored using a defect scale. The frailty index is calculated by dividing the total score of all items by 34. According to Saum *et al*. [11], the frailty index is divided into three levels: frailty (0.45 ≤ frailty index < 1), pre-frailty (0.20 < frailty index < 0.45), and non-frailty (0 < frailty index < 0.20).

The reliability of the frailty assessment scale was evaluated using Cronbach’s alpha coefficient. For the 34-item scale, Cronbach’s alpha was found to be 0.85, indicating a high level of internal consistency. Each subscale also demonstrated satisfactory reliability, with the health status variable, daily living ability scale, and instrumental daily living ability variable showing Cronbach’s alpha values of 0.82, 0.80, and 0.78 respectively. The chronic disease and clinical symptom variables showed Cronbach’s alpha values of 0.75 and 0.77, respectively. These values confirm the reliability of this tool for assessing frailty in patients.

### Baseline Characteristics of Epileptic Patients

We documented several baseline characteristics, including gender, age, body mass index (BMI), duration of disease, hypertension, diabetes and coronary heart disease for each study participant.

### Medication of Epilepsy Patients

The following data were recorded for each study participant: adherence to prescribed medication, duration of anti-epileptic drugs (≤10 years or >10 years), number of drugs taken (single-drug or multi-drug regimen), and type of anti-epileptic drugs (liver enzyme inducers or non-liver enzyme inducers).

### Prognosis of Elderly Patients with Epilepsy

In this study, patient seizures occurring within one year were recorded and classified based on the severity of the prognosis. Seizures were categorized into three groups: major seizure group (tonic-clonic), minor seizure group (absence, myoclonus, clonus, tonic, atonic, and partial seizures), and no seizure group. The classification of epilepsy followed the standard of epilepsy formulated by the Chinese Anti-Epilepsy Association in 2015 [8].

The 2015 classification standard established by the Chinese Anti-Epilepsy Association categorizes seizures based on their clinical features and electroencephalographic (EEG) patterns [8]. The classification includes:

**Focal seizures**: These seizures originate in one hemisphere of the brain and can be further classified into:

1. Focal-aware seizures: The person remains aware during the seizure.
2. Focal impaired awareness seizures: The person has impaired awareness during the seizure.
3. Focal to bilateral tonic-clonic seizures: Focal seizures that spread to both hemispheres, leading to generalized tonic-clonic seizures.

**Generalized seizures**: These involve both hemispheres of the brain from the onset and include:

1. Absence seizures: Characterized by brief lapses in awareness.
2. Myoclonic seizures: Involving sudden, brief, involuntary muscle jerks.
3. Clonic seizures: Characterized by rhythmic jerking movements.
4. Tonic seizures: Involving sudden muscle stiffening.
5. Atonic seizures: Characterized by sudden loss of muscle tone.
6. Tonic-clonic seizures: Involving a combination of tonic and clonic phases.

**Unknown onset seizures**: When the onset of the seizure is not observed or known.

The classification also considers the etiology (genetic, structural, metabolic, immune, infectious, or unknown causes), seizure type, and epilepsy syndrome, providing a comprehensive framework for diagnosing and managing epilepsy.

### Statistical Analysis

Statistical analysis was performed using SPSS 23.0 statistical software (IBM, Armonk, NY, USA). The normality of the data was assessed using the Shapiro-Wilk test. For normally distributed data, measurement data were expressed as mean ± standard deviation ($\bar{x}$ ± s) and one-way analysis of variance (ANOVA) was employed for multi-group comparison. Categorical data were expressed as absolute number, with inter-group comparison performed using the χ^2^ test. The Logistic regression model was used for multivariate analysis, and a *p*-value < 0.05 was considered statistically significant.

## Result

### Correlation Analysis between Clinical Features and Prognosis of Elderly Patients with Epilepsy

A correlation between baseline characteristics and prognosis is shown in Table 1. This study included 106 epileptic patients, including 56 males and 50 females, with an average age of 65.53 ± 2.03 years and an average BMI of 21.94 ± 3.52 kg/m^2^. Several clinical characteristics were significantly associated with the severity of seizures in elderly patients with epilepsy. Importantly, older individuals experienced more severe seizures (*p* < 0.001), with the mean age increasing as seizure severity increases. Furthermore, a longer duration of disease was linked to more severe seizures (*p* = 0.009), as was the presence of comorbid conditions such as diabetes (*p* = 0.002) and coronary heart disease (*p* < 0.001). Conversely, factors like gender, BMI, and hypertension did not show significant relationships with seizure severity (*p* = 0.752, *p* = 0.599, and *p* = 0.058, respectively).

**Table 1.** **Relationship between baseline characteristics and severity of seizures in elderly patients with epilepsy [$\bar{x}$ ± s, n (%)]**.

Variables		No seizures (n = 22)	Minor seizures (n = 62)	Major seizures (n = 22)	χ^2^/*F*-value	*p*-value
Gender					0.571	0.752
	Male	13	31	12		
	Female	9	31	10		
Age (years)		60.25 ± 2.94	65.97 ± 1.53	72.73 ± 2.21	207.500	<0.001
BMI (kg/m^2^)		21.63 ± 2.54	20.85 ± 3.13	21.06 ± 3.48	0.515	0.599
Duration of disease (years)					9.430	0.009
	≤10	14	25	4		
	>10	8	37	18		
Combined hypertension					5.689	0.058
	Yes	6	21	13		
	No	16	41	9		
Combined diabetes					12.640	0.002
	Yes	3	12	12		
	No	19	50	10		
Combined coronary heart disease					19.170	<0.001
	Yes	3	18	16		
	No	19	44	6		

Note: BMI, body mass index.

### Correlation Analysis between Frailty Index and Prognosis of Epilepsy Patients

We observed that the frailty index was significantly associated with the severity of seizures in epilepsy patients (*p* < 0.001). The non-frailty group exhibited a significantly lower incidence of major seizures, with only 1 patient experiencing major seizures. In contrast, the pre-frailty group had 11 patients with major seizures, and the frailty group had 10 patients with major seizures. Additionally, the frailty group had the highest number of patients with minor seizures (29 patients), indicating that elevated frailty is associated with higher seizure severity. A correlation between frailty index and prognosis is shown in Table 2.

**Table 2.** **Relationship between frailty index and seizure severity in epilepsy patients**.

Variables	Without seizures (n = 22)	Minor seizures (n = 62)	Major seizures (n = 22)	χ^2^-value	*p*-value
Non-frailty group (n = 29, 28.43%)	13	15	1	19.690	<0.001
Pre-frailty group (n = 35, 33.02%)	6	18	11		
Frailty group (n = 42, 39.62%)	3	29	10		

### Correlation Analysis between Medication Status and Prognosis of Patients

We found that the medication status was significantly associated with the severity of seizures. Patients who did not adhere to their medication regimen had a higher incidence of major seizures (*p* < 0.001). Additionally, the number of drugs used was significantly linked to seizure severity, with those on a single-drug regimen experiencing more severe seizures compared to those on multiple-drugs (*p* < 0.001). Conversely, the duration of medication and the type of drug did not show significant associations with the severity of seizures (*p* = 0.427 and *p* = 0.524, respectively). A correlation between the medication status and the extent of seizures is illustrated in Table 3.

**Table 3.** **The relationship between the medication status and the degree of seizures in epilepsy patients [n (%)]**.

Variables		Without seizures (n = 22)	Minor seizures (n = 62)	Major seizures (n = 22)	χ^2^-value	*p*-value
Take medicine regularly					22.740	<0.001
	Yes	19	21	5		
	No	3	41	17		
Medication duration (years)					1.701	0.427
	≤10	13	34	9		
	>10	9	28	13		
The number of drugs taken					14.840	<0.001
	Single-drug	4	38	15		
	Multi-drug	18	24	7		
Drug type					1.291	0.524
	Liver enzyme inducer	10	23	11		
	Non-liver enzyme inducer	12	39	11		

### Multivariate Logistic Analysis of Prognosis of Epilepsy Patients

Multivariate Logistic analysis in Table 4 indicated several significant predictors of seizure severity in elderly patients with epilepsy. A higher frailty index (*p* = 0.033, odds ratio (OR) = 1.362, 95% confidence interval (CI): 1.025–1.810), age over 70 years (*p* = 0.015, OR = 5.954, 95% CI: 1.418–24.996), longer disease duration (*p* = 0.003, OR = 2.052, 95% CI: 1.284–3.280), and the presence of coronary heart disease (*p* < 0.001, OR = 5.078, 95% CI: 4.069–6.337) were all significantly associated with more severe seizures. Regular medication use demonstrated a significant relationship (*p* = 0.022, OR = 1.073, 95% CI: 1.010–1.141). Furthermore, diabetes and the number of drugs taken did not show significant associations (*p* > 0.05).

**Table 4.** **Multi-factor correlation analysis of the severity of epileptic seizures in the elderly**.

Variables	β	S.E.	Wald χ^2^	*p*-value	OR	95% CI
Frailty index	0.309	0.145	4.541	0.033	1.362	1.025–1.810
Age (>70 years)	1.784	0.732	5.939	0.015	5.954	1.418–24.996
Duration of disease	0.719	0.239	9.017	0.003	2.052	1.284–3.280
Combined diabetes	0.081	0.224	0.131	0.716	1.085	0.700–1.682
Combined coronary heart disease	1.625	0.113	206.799	<0.001	5.078	4.069–6.337
Take medicine regularly	0.071	0.031	5.245	0.022	1.073	1.010–1.141
The number of drugs taken	3.079	1.892	2.650	0.103	21.73	0.523–886.53

Note: S.E., standard error; CI, confidence interval; OR, odds ratio.

## Discussion

Epidemiological studies have shown that older people have the highest incidence and prevalence of epilepsy among all age groups [12, 13]. Compared to younger patients, elderly patients are more likely to experience focal seizures, have a longer duration of post-seizure confusion, and frequently face epileptic status [14]. The annual incidence in patients aged 60 years and older is reported to be 86 per 100,000, almost double that of the general population, and it increases even higher after age 70 [15]. Mortality rates also increase with age, etiology, and duration of the condition. Generally, the mortality rate for epilepsy in the elderly is 38%, but it increases to nearly 50% for those over 80 years of age [16]. This age group differs in clinical presentations and etiologies compared to younger individuals, with a substantial proportion of patients having comorbidities and cognitive impairments, making diagnosis and treatment extremely challenging. Despite the complexity of diagnosis and treatment of elderly epilepsy patients, few clinical and basic studies have been conducted on this population.

This study shows a strong link between the frailty index and the severity of epilepsy in patients. With the aging of the population, the impact of the frailty index on epilepsy needs to be explored. Frailty is a decline in overall health and ability to function independently, increasing the likelihood of further deterioration. It covers a wide range of subclinical health, including functional status, symptoms, and disabilities, providing a more comprehensive understanding of a patient’s health. A cross-sectional study that initially explored the frailty condition in a small number of elderly epilepsy patients showed that those with higher body weight had reduced tolerance to anti-epileptic drugs [17].

Current studies have reported a strong association between frailty and certain neurodegenerative diseases, such as Alzheimer’s disease, with elderly patients who have a higher frailty index being eight times more likely to suffer from dementia [18, 19, 20]. However, there is limited research on the association between frailty index and epilepsy in elderly patients. Another study assessing the frailty index in adults over 60 years found that older people with epilepsy had a higher degree of frailty [21], which aligns with the results of this study, indicating that the frailty index is highly correlated with the prognosis of epilepsy patients.

Age, duration of disease, diabetes, and coronary heart disease were found to be substantially correlated with prognosis, with the difference being statistically significant (*p* < 0.05). The reasons for the age-related severity of epilepsy in the elderly may involve the elevated chance of conditions such as stroke, head injury from falls, brain tumors, and other brain diseases with the growth of age [22, 23], all being identified as risk factors for the onset of epilepsy. Therefore, epilepsy often co-exists with a variety of neurological diseases, including neurodegenerative diseases [24]. Furthermore, the duration of epilepsy plays a vital role in its prognosis. Numerous studies have shown that the longer the duration of epilepsy, the more severe the impairment of patients’ memory, naming ability, lexical breadth, attention, and executive function [25, 26]. The persistence of epilepsy may reduce the cognitive function of patients, and the recurrence rate of patients with a long course of disease is higher than that of patients with a short course of disease [27]. Hypertension is a non-cerebral comorbidity associated with epilepsy, and the activation of the renin-angiotensin-aldosterone system can aggravate and stimulate epileptic seizures.

Consequently, antihypertensive drugs that inhibit the renin-angiotensin-aldosterone system may also have a multi-effect anti-epileptic therapeutic effect [28]. Multiple studies and case reports have documented the co-occurrence of epilepsy and hypertension, with some studies indicating that hypertension is an independent predictor of late-onset epilepsy, regardless of vascular injury [29, 30]. Diabetes is also a significant risk factor for a poor epilepsy prognosis. Studies have indicated a close link between the incidence of epilepsy and type 2 diabetes mellitus (T2DM), which may increase the risk of epilepsy independent of severe hypoglycemia [31, 32, 33]. A study indicated a 50% increased risk of epilepsy in T2DM patients across all gender and age groups [34]. The underlying mechanism may include abnormal blood glucose levels disrupting the neuronal inhibition and excitation balance, thereby triggering focal motor seizures [35]. Coronary heart disease is a common chronic condition in the elderly that is strongly corelated with epilepsy, with a complex and multifaceted pathophysiological mechanism. Generally, people with epilepsy may experience reduced physical activity, unhealthy diet, and increased levels of stress, anxiety, and depression, all of which can affect a healthy lifestyle and promote coronary heart disease. Additionally, anti-epileptic drugs may cause weight gain and elevated cholesterol levels, potentially increasing atherosclerotic burden [36]. Epilepsy is also a sign of vascular diseases, leading to a recent concept of “epileptic heart” in the medical community. Most of the research in this area focuses on the relationship between sudden cardiac death and epilepsy, underscoring the significance of preventing cardiovascular diseases in patients with epilepsy [37].

This study also found that regular medication adherence, the number of doses and prognosis are interrelated. In the elderly, cerebrovascular disease is the most common cause of epilepsy, often accompanied by other conditions such as hypertension, heart disease, diabetes, and hyperlipidemia. As a result, these patients commonly take additional medications for diseases such as hypertension and diabetes, which can result in drug interactions and adverse therapeutic reactions when combined with anti-epileptic drugs. It presents the unique challenge of anti-epileptic drug therapy in elderly patients with epilepsy [38].

Therefore, to address these challenges, it is crucial for neurologists and physicians from other specialties to collaborate in devising an individualized medication plan. This plan should not only ensure effective control of underlying diseases such as hypertension, diabetes, and coronary heart disease but also minimize the risk of drug interactions. Selecting the lowest effective dose of anti-epileptic drugs is crucial for controlling seizures. Additionally, patient compliance should be strengthened, and the patient should be instructed to take medicine regularly to avoid missing or undertaking drugs. Because the number of epilepsy drugs used depends on the half-life of the drug, it is necessary to maintain a certain blood concentration of drugs in the body for a long time, so as to effectively improve the prognosis of patients and reduce the seizure of elderly patients.

Furthermore, this study addresses a significant gap in the existing literature by investigating the complex relationship between frailty index, clinical characteristics, anti-epileptic drug use, and prognosis in elderly patients with epilepsy. While previous studies have explored certain aspects of epilepsy in the elderly separately, our study uniquely integrates these variables to provide a comprehensive understanding of their combined effects on patient outcomes. By employing a robust retrospective analysis, we have identified crucial factors that significantly influence the severity of seizures, offering valuable insights for improving clinical management and patient care strategies in this vulnerable population. However, as a retrospective study, our research has several limitations. First, the retrospective design relies on existing medical records, which may introduce biases due to incomplete or inaccurate documentation. Second, the limited sample size may restrict the generalizability and representativeness of the results. While our multivariate Logistic regression analysis revealed multiple factors influencing the severity of seizures, the influence of other unmeasured confounding factors cannot be determined. Additionally, this study only focused on the effects of medication adherence and the number of drugs on seizure severity without delving into the specific impacts of different types of anti-epileptic drugs. Future research should aim to design prospective studies with larger sample sizes to further validate our findings. Additionally, a more in-depth investigation into the specific effects of different anti-epileptic drugs and their impact on the prognosis of elderly epilepsy patients is warranted to develop more precise and personalized treatment strategies.

## Conclusion

In summary, we examined the correlation between frailty index, clinical features, drug use, and clinical prognosis in elderly patients with epilepsy and found that frailty index, age, disease duration, coronary heart disease, and regular medication are highly correlated with the prognosis. These findings provide a crucial clinical basis for accurate clinical diagnosis and treatment. Providing individualized diagnosis and precise treatment can further improve the prognosis of elderly patients with epilepsy.

## Availability of Data and Materials

The data used to support the findings of this study are available from the corresponding author upon request.
